# An examination of the associations between nutritional peaking strategies in physique sport and competitor characteristics

**DOI:** 10.1080/15502783.2024.2377178

**Published:** 2024-07-15

**Authors:** Kai A. Homer, Matt R. Cross, Eric R. Helms

**Affiliations:** a Auckland University of Technology, Sport Performance Research Institute New Zealand (SPRINZ), Auckland, New Zealand; b Florida Atlantic University, Department of Exercise Science and Health Promotion, Muscle Physiology Laboratory, Boca Raton, FL, USA

**Keywords:** Peak week, nutrition, carbohydrate, water, sodium

## Abstract

**Background:**

Physique athletes are subjectively judged on their on-stage esthetic per their competition division criteria. To succeed, competitors look to acutely enhance their appearance by manipulating nutritional variables in the days leading up to competition, commonly referred to as peak week (PW). Despite their documented wide adoption, PW strategies lack experimental evidence. Further, the relationship between the specific strategies and the characteristics of the competitors who implement them are unknown. The aim of this research was to examine the effect of competitor characteristics on the specific nutritional peaking strategies implemented, the length of these strategies, and the range of daily carbohydrate (CHO) intakes during these strategies.

**Methods:**

A 58-item survey was developed to gather information on peak week nutrition and training practices of physique athletes. A total of 160 respondents above the age of 18 who had competed in the last 5 years completed the nutrition section. The topics analyzed for this paper included competitor demographics, peaking strategies utilized, and PW CHO intakes. Competitor demographics are presented with the use of descriptive statistics. Associations between competitor demographics and peaking strategies implemented, peaking strategy length, and daily CHO intake ranges were assessed using multiple logistic regression, multiple ordinal logistic regression, and linear mixed models, respectively.

**Results:**

From the sampled population, ages 24–39 years (71.2%), male (68.8%), natural (65%), and amateur (90%) were the most common characteristics from their respective categories, while mean competition preparation length was 20.35 ± 8.03 weeks (Males: 19.77 ± 7.56 weeks, Females: 21.62 ± 8.93 weeks), competition preparation body mass loss was 11.5 ± 5.56 kg (M: 12.7 ± 5.76 kg, F: 7.16 ± 3.99 kg), and competition body mass was 72.09 ± 15.74 kg (M: 80.15 ± 11.33 kg, F: 54.34 ± 7.16 kg). For males, the highest and lowest daily CHO intake during PW were 489.63 ± 224.03 g (6.22 ± 2.93 g/kg body mass) and 148.64 ± 152.01 g (1.94 ± 2.17 g/kg), respectively, while for females these values were 266.73 ± 131.23 g (5.06 ± 2.67 g/kg) and 94.42 ± 80.72 g (1.81 ± 1.57 g/kg), respectively. CHO back loading (45%) and water loading (40.6%) were the most popular peaking strategies, while the most prevalent peaking strategy length was 7 days (27.2%). None of the competitor characteristics predicted the use of CHO-based peaking strategies nor peaking strategy length. For non-CHO-based strategies, drug-enhanced competitors were more likely to restrict water than non-drug enhanced, while males and professional competitors had greater odds of loading sodium than females and amateurs, respectively. Finally, when comparing the disparity in highest and lowest CHO intakes during peak week, sex was the only significant factor.

**Conclusions:**

The results of this survey provide further information on the nutritional peaking strategies implemented by competitors. Certain characteristics were identified as predictors of sodium loading and water restriction, and the range of daily PW CHO intake. Contrastingly, no associations were found for CHO-based peaking strategies or peaking strategy length. While our analyses may be underpowered, and thus results should be interpreted with caution, it appears the nutritional peaking strategies implemented by physique competitors are seemingly complex and highly individual.

## 1. Background

Physique sport involves the subjective judging of competitors on variables such as muscle size, symmetry, and body fat levels that are presented through posing [1,2]. Competition preparation refers to the pre-contest period, where prolonged energy restriction and training manipulations are implemented by competitors to best fulfil the judging criteria of their division [1,2]. To this end, physique athletes acutely manipulate their nutrition and training in the week prior to competition, which is colloquially known as peak week (PW) [1,2]. During PW, some athletes attempt to acutely enhance muscle size and definition by increasing intramuscular glycogen and its associated water while minimizing subcutaneous layer thickness by manipulating carbohydrate (CHO) intake, electrolytes, and water [3]. These practices are rationalized by the high concentration of sodium and potassium in extracellular and intracellular compartments, respectively, and the actions of the sodium-potassium pump in regulating cell volume [1,4]. Thus, it has been proposed by physique athletes and authors that loading potassium while reducing sodium alters the cellular concentrations of these ions, which when combined with increased muscle glycogen causes water to be drawn into muscle via an osmotic gradient [1,2]. Whilst expanding, the body of scientific evidence regarding these practices remain limited.

Despite little experimental evidence supporting their efficacy, PW strategies are common and have been documented in single-subject case studies [5–9], group-level observational studies [10–12], and compared in a quasi-experimental design [13]. Further, nutritional PW practices of physique athletes have been described in larger samples with cross-sectional data. Specifically, Chappell and Simper [14] reported that some form of CHO manipulation was practiced by 91% of their sample of 81 drug-free (i.e. natural) British bodybuilders, with restriction typically followed by loading to induce muscle glycogen supercompensation. Additionally, both water loading and subsequent restriction was implemented by 25% of respondents, and sodium loading by 18.5%. Qualitative interviews of experienced, high-level male bodybuilders conducted by Mitchell et al. [15] revealed that CHO loading strategies were adopted by six of the seven participants. More recently, in a survey of 29 physique athletes, the majority reported implementing CHO and water manipulation; however, the strategies as well as the reported macronutrient and water intakes were highly varied [16]. To further complicate interpretation, participants were participating in competitions where performance-enhancing drugs (PEDs), such as anabolic-androgenic steroids (AAS), are not tested for. Competitors who use PEDs (commonly referred to as being “enhanced”) are prevalent in such competitions [17–19], and the profound physiological effects of PED use plausibly impacts the outcomes of nutritional PW strategies. Therefore, competitor characteristics such as drug-use status must be considered in analyses and when interpreting outcomes.

While existing cross-sectional examinations hint to the prevalence of nutritional peaking strategies, small and homogenous samples limit the certainty of any reported associations and subsequent conclusions. Sampling from a broader population of physique athletes with the exploration of factors such as age, sex, and drug-use status should bolster the growing understanding of nutritional PW strategies. The aim of this research was to explore relationships between competitor characteristics and (1) peaking strategy implemented, (2) length of strategies, and (3) range of daily CHO intakes during PW.

## 2. Methods

### 2.1. Design

This research employed a cross-sectional survey to capture the dietary and training practices of physique athletes during PW (although the present study focuses solely on dietary practices), with all questions relating to their last competition. An anonymous survey developed using Qualtrics (Qualtrics, Provo, UT) was distributed via social media. The first draft of the survey was developed by the research team. Subsequent drafts of the survey were piloted by a professional bodybuilder who actively conducts sport science research. Feedback from the piloting was used to modify the wording of certain questions and to add or remove questions altogether. The survey was available from August to October 2022. The outcome data were used to characterize the specific nutrition practices of physique athletes during PW, including their employed peaking strategies.

### 2.2. Participants

A total of 160 physique athletes (110 males, 50 females) completed the nutrition section of the survey (see Table 1). All participants were made aware of the research aims and methods through the participant information sheet at the beginning of the survey and acknowledged that by taking the survey they were providing informed consent. All participants were required to be aged 18 years or older and have competed in at least one physique competition in the last 5 years. While the education level of the participants was not captured, the survey employed terminology familiar to competitive physique athletes, as well as providing full definitions of terms where appropriate. This research was approved by the Auckland University of Technology Ethics Committee (AUTEC reference number 22/208).

**Table 1.** Sample characteristics.

Age (years)	Frequency	Percentage	
18–23	22	13.8%	
24–39	114	71.2%	
40–49	16	10%	
50–59	8	5%	
60+	0	0%	
**Sex**			
Male	110	68.8%	
Female	50	31.2%	
**Drug Status**			
Natural	104	65%	
Enhanced	56	35%	
**Competitor Level**			
Amateur	144	90%	
Professional	16	10%	
**Competitor Division**			
Men’s Bodybuilding	57	39.3%	
Classic Physique	18	12.4%	
Men’s Physique	26	17.9%	
Bikini	25	17.2%	
Other Women’s Combined	19	13.1%	
Missing/undisclosed	15	9.4%	
	Frequency (M, F)	M Mean ± SD	F Mean ± SD
Competition Preparation Length (weeks)	160 (110, 50)	19.77 ± 7.56	21.62 ± 8.93
Competition BM (kg)		80.15 ± 11.33	54.34 ± 7.16
∆BM (kg)		12.7 ± 5.76	7.16 ± 3.99
Highest PW Daily CHO Intake (g) [Relative to BM (g/kg)]	91 (65, 26)	489.63 ± 224.03 [6.22 ± 2.93]	266.73 ± 131.23 [5.06 ± 2.67]
Lowest PW Daily CHO Intake (g) [Relative to BM (g/kg)]		148.64 ± 152.01 [1.94 ± 2.17]	94.42 ± 80.72 [1.81 ± 1.57]

*Abbreviations and definitions: SD* = standard deviation, Natural = PED-free competitor, Enhanced = competitor using PED(s), *M* = male, F = female, BM = body mass, ∆BM = change in body mass during competition preparation, PW = peak week, CHO = carbohydrate.

### 2.3. Procedures

The 58-item survey was divided into competitor information, nutrition, and training sections. All questions were relevant to the respondent’s most recent competition. The competitor information section served to gather descriptive participant data including age, sex, drug-use status (either natural or enhanced, defined as not using or using PEDs including AAS, prescription diuretics, growth hormone, insulin, fat loss agents such as clenbuterol or dinitrophenol, or any other substance banned by their respective bodybuilding federation in the case of tested competitors, respectively), competitor level (amateur or professional) and division (e.g. men’s bodybuilding, bikini), competition preparation length, change in body mass during competition preparation (∆BM), and competition body mass (BM). The age categories of 18–23, 24–39, 40–49, 50–59, and 60+ years were selected to represent the commonly used teen, open, masters, grandmasters, and over 60’s age classes, respectively. Questions in the nutrition section were presented in the following order: peaking strategies utilized, macronutrient and micronutrient intakes and food sources during PW, competition preparation energy and CHO intakes not during PW, and competition day strategies. Competitor descriptives, peaking strategies, and PW CHO intakes were analyzed for this paper.

### 2.4. Data acquisition and preparation

All questions were designed to maintain the anonymity of participants, and no identifying information was present in the Qualtrics output. Data were initially screened for insufficiently complete and unambiguously false responses. Incomplete responses were determined by an incomplete competitor information section, while any missing data from the nutrition section was omitted. All adequate responses to the nutrition section were then organized and coded for analysis.

As the outcome variables of interest, responses to each peaking strategy (i.e. whether the strategy was implemented or not) were coded as binary outcomes for subsequent analyses. The CHO-based peaking strategies examined were back loading, mid loading, front loading, linear loading, and CHO restriction, while non-CHO strategies were fat loading, water loading, water restriction, sodium loading, sodium restriction, and potassium loading. These variables are defined in the supplementary material. In addition to selecting the peaking strategy that competitors implemented, an option to qualitatively describe the strategy was also presented. Responses to peaking strategy length were treated as ordinal values, while the highest and lowest daily CHO intakes were extracted to make within-participant comparisons of daily PW CHO intake ranges. Relative CHO intake was determined by dividing the absolute values by competition BM.

The categorical predictors for all models were age group, sex, level, and drug-use status. The 24–39 age group, male, amateur, and natural competitors were set as the reference groups for each corresponding variable. These groups were selected as they had the largest N relative to other groups. The other levels of age consisted of 18–23, 40–49, and 50–59-years, while no responses for the 60+ category were recorded. Additionally, competition preparation length (weeks), competition BM, and ∆BM were included as continuous predictors. For competitor division, all non-bikini female competitors were combined into one group due to the dispersion of responses across similar divisions.

### 2.5. Statistical analysis

All statistical analyses were performed in R language and environment for statistical computing [20] using the lme4 [21], and emmeans [22] packages, with the performance [23] package used to assess model diagnostics. The supplementary files, dataset analyzed, and the R scripts which detail model specifications and diagnostics are available at the Open Science Framework repository (https://osf.io/9ghfz).

Frequencies and percentages were obtained for categorical competitor descriptives, peaking strategies, and length of peaking strategy, while means and standard deviations were calculated for competition preparation length, competition BM, ∆BM, and highest and lowest daily CHO intakes during PW.

To fulfil our first research aim, a series of multiple logistic regression analyses was conducted to explore relationships between each peaking strategy utilized and participant demographics. Predictors included the categorical variables described previously, and competition preparation length and ∆BM. Model fit was assessed using likelihood ratio tests (LRT), Tjur’s coefficient of determination (*R*^*2*^), and binned residual plots with all reductions to the structure of the models made on theoretical grounds to obtain the best fit while satisfying assumptions and maintaining model parsimony. Within each multiple logistic regression model, odds ratios with 95% CI for each predictor variable are presented.

To address our second research aim and provide further context on the specifics of peaking strategies, multiple ordinal logistic regression was conducted to discern any relationships between competitor characteristics and peaking strategy length. The Brant–Wald test was used to assess the parallel regression assumption and reduce the model structure in conjunction with the Akaike Information Criterion score (AIC), deviance statistic, and LRT to obtain best fit while satisfying assumptions. For each of the predictor variables within the multiple ordinal logistic regression model, odds ratios and 95% CIs were calculated.

Linear mixed-effects models were used to examine the relationship between the highest and lowest daily CHO intakes (both absolute and relative to BM) during PW and all predictors described previously. For both models, participants were treated as random effects to control for the general variation and non-independence of data. Restricted maximum likelihood estimation was used to determine both random and fixed effect regression parameters (*β*). The best model fit, and the contribution effects were obtained by comparing explored models using the LRT, AIC, and deviance statistic. The statistical significance of predictor variables was derived using t-tests based on the Satterthwaite approximation, and the Holm-Bonferroni correction was applied post-hoc to the categorical variable age as it contained more than two levels. *β* with 95% CI are presented for both linear mixed models. An *α* level of .05 was set for all regression models and LRTs.

## 3. Results

### 3.1. Sample characteristics

Of the 160 responses to the nutrition section of the survey, the proportion of males (68.8%) was greater than females (31.2%). For the other categorical descriptives of the survey, more natural competitors (65%) responded to the survey than enhanced (35%) and more amateur (90%) than professional (10%). For descriptors with more than two levels, ages 24–39 years (71.2%) and men’s bodybuilding (39.3%) made up the highest proportion of respondents. Mean competition preparation length was 20.35 ± 8.03 weeks (Males: 19.77 ± 7.56 weeks, Females: 21.62 ± 8.93 weeks), total competition preparation change in body mass was 11.50 ± 5.56 kg (M: 12.7 ± 5.76 kg, F: 7.16 ± 3.99 kg), and mean competition body mass was 72.09 ± 15.74 kg (M: 80.15 ± 11.33 kg, F: 54.34 ± 7.16 kg). Finally, the highest and lowest daily CHO intake during PW in males were 489.63 ± 224.03 g (6.22 ± 2.93 g/kg body mass) and 148.64 ± 152.01 g (1.94 ± 2.17 g/kg), respectively, while for females these values were 266.73 ± 131.23 g (5.06 ± 2.67 g/kg) and 94.42 ± 80.72 g (1.81 ± 1.57 g/kg), respectively. The characteristics of our sample are summarized in Table 1.

From the peaking strategies, CHO back loading (45%), water restriction (40.6%), and water loading (40.6%) were the most utilized while CHO restriction (6.9%), potassium loading (6.9%) and CHO front loading (11%) were the least prevalent. For length of peaking strategy, starting 7 days before competition was most popular (27.2%), whereas no competitor started their strategy exactly 13 days before competition (Table 2).

**Table 2.** Frequencies of peaking strategies utilized and peaking strategy length.

	N	Percentage
Peaking Strategy		
No Strategy	12	7.5%
Carbohydrate Back Load	72	45%
Carbohydrate Mid Load	30	18.8%
Carbohydrate Front Load	18	11.2%
Carbohydrate Linear Load	33	20.6%
Carbohydrate Restriction	11	6.9%
Fat Load	34	21.2%
Water Load	65	40.6%
Water Restriction	56	35%
Sodium Load	42	26.2%
Sodium Restriction	26	16.2%
Potassium Load	11	6.9%
Other	8	5%
**Peaking Strategy Length (days)**		
1	4	2.7%
2	7	4.8%
3	6	4.1%
4	6	4.1%
5	22	15%
6	20	13.6%
7	40	27.2%
8	16	10.9%
9	1	0.7%
10	9	6.1%
11	1	0.7%
12	2	1.4%
13	0	0%
14	13	8.8%

Definitions of each peaking strategy are presented in the supplementary material.

### 3.2. Associations between peaking strategies and sample characteristics

When examining the association between peaking strategy implemented and competitor characteristics, only water restriction (*χ*^*2*^ = 16.07, df = 9, *p* < .05) and sodium loading (*χ*^*2*^ = 15.92, df = 9, *p* < .05) were significant and explained 9.3% and 10.3% variance in the outcome variables, respectively. Being an enhanced competitor was a significant predictor for restricting water in comparison to being a natural competitor (OR = 2.49 [1.17, 5.38], *p* < .05), while males (OR = 2.99 [1.19, 8.14], *p* < .05) and professionals (OR = 4.42 [1.19, 17.97], *p* < .05) had higher odds of loading sodium than their female and amateur counterparts, respectively. All results for the multiple logistic regression models for the CHO-based strategies are shown in Table 3. Table 4 displays all other strategies.

**Table 3.** Factors affecting the implementation of carbohydrate-based peaking strategies.

	Back Load			Mid Load			Front Load			Linear Load			CHO Restriction		
Predictors	*Odds Ratios* *(95% CI)*		*p*	*Odds Ratios* *(95% CI)*		*p*	*Odds Ratios* *(95% CI)*		*p*	*Odds Ratios* *(95% CI)*		*p*	*Odds Ratios* *(95% CI)*		*p*
(Intercept)	1.32 (0.41, 4.3)		.646	0.06 (0.01, 0.35)		**<.001**	0.08 (0.01, .51)		.**01**	0.45 (0.1, 1.87)		.276	0.63 (0.07, 5.16)		.669
Age [18–23]	0.78 (0.29, 2.04)		.616	1.44 (0.41, 4.46)		.54	0.9 (0.13, 3.93)		.898	0.16 (0.001, 0.87)		.087	1.37 (0.18, 7.25)		.726
Age [40–49]	0.83 (0.18, 3.75)		.811	0.8 (0.14, 3.3)		.769	1.57 (0.28, 6.83)		.572	1.03 (0.24, 3.69)		.97			
Age [50–59]	1.29 (0.24, 7.04)		.767	0.6 (0.03, 3.88)		.648				1.02 (0.14, 4.92)		.986	1.89 (0.09, 14.38)		.59
Sex [Female]	0.74 (0.34, 1.57)		.433	0.76 (0.27, 2.01)		.594	0.98 (0.27, 3.22)		.969	0.87 (0.35, 2.09)		.753	0.95 (0.2, 3.99)		.95
Level [Professional]	0.44 (0.11, 1.5)		.211	1.65 (0.37, 6.53)		.487	2.11 (0.37, 9.76)		.36	0.94 (0.22, 3.34)		.932	1.26 (0.06, 10.23)		.848
Drug Status [Enhanced]	1.56 (0.76, 3.22)		.266	1.57 (0.62, 3.95)		.34	1.98 (0.66, 5.91)		.215	0.76 (0.3, 3.34)		.545	0.61 (0.11, 2.56)		.516
Competition Preparation Length (weeks)	1 (0.96, 1.04)		.986	1.07 (1.01, 1.13)		.**025**	1.05 (0.91, 1.05)		.606	1.02 (0.97, 1.08)		.393	0.96 (0.86, 1.04)		.36
∆Body Mass (kgs)	0.97 (0.9, 1.03)		.321	1.01 (0.93, 1.1)		.743	1.02 (0.94, 1.15)		.422	0.94 (0.85, 1.02)		.139	0.89 (0.76, 1.03)		.128
Likelihood Ratio Test *p < .05 indicates significant difference from null model*	*χ*^*2*^	df		*χ*^*2*^	df		*χ*^*2*^	df		*χ*^*2*^	df		*χ*^*2*^	df	
	7.17	9	.518	7.59	9	.475	4.12	8	.766	6.67	8	.464	3.45	8	.84
Tjur’s *R*^*2*^	.044			.05			.023			.042			.042		
Binned Residuals *>95% indicates good model fit*	100%			92%			67%			92%			50%		

Abbreviations: CI = confidence interval, ∆Body Mass = change in body mass during competition preparation, Likelihood Ratio Test = assessment of model fit in comparison to the null model, χ^2^ = chi-squared statistic, df = degrees of freedom, Tjur’s *R*^*2*^ = Tjur’s coefficient of determination/discrimination, assessment of model predictive power, Binned Residuals = assessment of model fit. Odds ratios and *p*-values for the variable in square brackets are calculated in comparison to the corresponding reference group. Bold text denotes statistical significance.

**Table 4.** Factors affecting the implementation of non-carbohydrate-based peaking strategies.

	Fat Load			Water Load			Water Restriction			Sodium Load			Sodium Restriction			Potassium Load		
Predictors	*Odds Ratios* *(95% CI)*		*p*	*Odds Ratios* *(95% CI)*		*p*	*Odds Ratios* *(95% CI)*		*p*	*Odds Ratios* *(95% CI)*		*p*	*Odds Ratios* *(95% CI)*		*p*	*Odds Ratios* *(95% CI)*		*p*
(Intercept)	0.59 (0.14, 2.43)		.464	0.54 (0.15, 1.86)		.331	0.65 (0.19, 2.26)		.501	0.23 (0.05, 0.9)		.**038**	0.07 (0.01, 0.36)		.**002**	0.08 (0.01, 0.7)		.**031**
Age [18–23]	0.4 (0.06, 1.59)		.25	1.78 (0.66, 4.78)		.25	2.17 (0.8, 5.97)		.127	0.66 (0.17, 2.08)		.507	1.83 (0.45, 6.42)		.358	0.87 (0.04, 5.82)		.903
Age [40–49]	1.54 (0.36, 5.73)		.531	0.63 (0.17, 2.08)		.459	0.64 (0.29, 3.81)		.897	0.29 (0.05, 1.27)		.133	0.71 (0.1, 3.39)		.699	0.62 (0.03, 4.57)		.689
Age [50–59]	2.12 (0.4, 9.72)		.34				0.64 (0.09, 3.19)		.615	1.58 (0.3, 7.17)		.558	0.77 (0.04, 5.03)		.815			
Sex [Female]	1.22 (0.49, 3)		.667	1.55 (0.71, 3.41)		.269	2.04 (0.9, 4.69)		.088	0.33 (0.12, 0.84)		.**026**	3.67 (1.36, 10.39)		.**011**	1.72 (0.42, 6.84)		.435
Level [Professional]	0.42 (0.06, 1.88)		.309	0.81 (0.22, 2.72)		.734	0.47 (0.09, 1.81)		.306	4.42 (1.19, 17.97)		.**029**	0.3 (0.01, 2.02)		.297	0.82 (0.04, 6.45)		.869
Drug Status [Enhanced]	1.6 (0.67, 3.78)		.283	1.9 (0.9, 4.08)		.095	2.49 (1.17, 5.38)		.**019**	1.98 (0.87, 4.57)		.106	3.09 (1.16, 8.56)		.**025**	2.88 (0.74, 11.79)		.125
Competition Preparation Length (weeks)	0.97 (0.92, 1.03)		.357	1.02 (0.97, 1.07)		.469	0.96 (0.91, 1.01)		.122	1.05 (0.99, 1.1)		.079	1.01 (0.95, 1.08)		.702	1.02 (0.93, 1.12)		.641
∆BM (kgs)	0.96 (0.88, 1.04)		.343	0.95 (0.88, 1.02)		.157	1 (0.93, 1.07)		.967	0.96 (0.89, 1.03)		.255	0.98 (0.9, 1.07)		.729	0.91 (0.79, 1.03)		.146
Likelihood Ratio Test *p < .05 indicates significant difference from null model*	*χ*^*2*^	df		*χ*^*2*^	df		*χ*^*2*^	df		*χ*^*2*^	df		*χ*^*2*^	df		*χ*^*2*^	df	
	9.9	9	.272	7.51	8	.377	16.07	9	.**041**	15.92	9	.**044**	12.59	9	.127	5.79	8	.564
Tjur’s *R*^*2*^	.065			.048			.093			.103			.088			.038		
Binned Residuals *>95% indicates good model fit*	100%			92%			92%			100%			77%			58%		

Abbreviations: CI = confidence interval, ∆BM = change in body mass during competition preparation, Likelihood Ratio Test = assessment of model fit in comparison to the null model, χ^*2*^ = chi-squared statistic, df = degrees of freedom, Tjur’s *R*^*2*^ = Tjur’s coefficient of determination/discrimination, assessment of model predictive power, Binned Residuals = assessment of model fit. Odds ratios and *p*-values for the variable in square brackets are calculated in comparison to the corresponding reference group. Bold text denotes statistical significance.

There was no significant relationship between the included predictor variables and peaking strategy length (*χ*^*2*^ = 2.35, df = 3, *p* > .05). The models examining factors influencing daily CHO intake ranges during peak week (Table 5) explained 58.2% and 62.4% of the variance in absolute and relative differences, respectively. Sex was the only significant predictor for PW CHO intake, where the disparity between the lowest and highest PW CHO intakes was smaller in females compared to males for absolute intake (*β* = −108.8 [−216.3, −1.2], *p* < .05). When CHO intake was relative to body mass, again, only the effect of sex was significant (*β* = −1.7 [−3.2, −0.2], *p* < .05).

**Table 5.** Factors affecting the (1) the absolute difference between the lowest and highest daily carbohydrate intake during peak week; (2) the relative difference between the lowest and highest daily carbohydrate intake during peak week; and (3) peaking strategy length.

Predictors	Factors Affecting Absolute CHO Difference		Factors Affecting Relative CHO Difference			Factors Affecting Peaking Strategy Length		
	*β (95% CI)*	*p*	*β (95% CI)*		p	*Odds Ratios* *(95% CI)*		p
(Intercept)	273.4 (59.9, 486.8)	.**012**	9.7 (5.7, 13.7)		**<.001**			
CHO Intake [Lowest]	−292.8 (−338.5, −247.1)	**<.001**	−3.9 (−4.6, −3.4)		**<.001**			
Age [18–23]	12 (−85.4, 109.4)	1*	0.2 (−1.2, 1.6)		1*			
Age [40–49]	38.9 (−65.4, 143.1)	1*	0.6 (−0.9, 2.1)		1*			
Age [50–59]	−23.9 (−152.5, 104.6)	1*	−0.4 (−2.2, 1.5)		1*			
Sex [Female]	−108.8 (−216.3, −1.2)	.**047**	−1.7 (−3.2, −0.2)		.**031**	0.9 (0.5, 1.8)		.803
Level [Professional]	−5.8 (−107.7, 96)	.911	−0.3 (−1.7, 1.2)		.691			
Drug Status [Enhanced]	−69.7 (−148.7, 9.18)	.083	−1 (−2.1, 0.1)		.075	0.6 (0.3, 1.2)		.131
Contest Preparation Length (weeks)	−1.1 (−5.2, 3)	.602	−0.02 (−0.1, 0.04)		.454			
∆BM (kg)	5.57 (−0.14, 11.3)	.056	0.1 (−0.02, 0.2)		.110	0.99 (0.9, 1.1)		.813
Competition BM (kg)	0.8 (−2.7, 4.2)	.665	−0.04 (−0.09, 0)		.072			
**Random Effects**								
σ^2^	24344.85			3.98				
τ_00 Participant_	6943.45			1.85				
ICC	.22			.32				
N	91			91		147		
Observations	182			182		147		
Likelihood Ratio Test *p < .05 indicates significant difference from null model*						*χ*^*2*^	df	
						2.35	3	.504
Marginal *R*^*2*^ /Conditional *R*^*2*^	.463/.582			.449/.624				
*R*^*2*^ Nagelkerke						.016		

Abbreviations: CHO = carbohydrate, *β* = regression parameter estimate of fixed effects, CI = confidence interval, ∆Body Mass = change in body mass during competition preparation, σ^2^ = variance of error, τ_00 Participant_ = random effects of participants, ICC = Intraclass correlation coefficient, random effects: total variance ratio, Likelihood Ratio Test = assessment of model fit in comparison to the null model, χ^*2*^ = chi-squared statistic, df = degrees of freedom, *R*^*2*^ = coefficient of determination, Marginal *R*^*2*^ = proportion of variance from fixed effects, Conditional *R*^*2*^ = proportion of variance from both fixed and random effects, *R*^*2*^ Nagelkerke = proportion of variance explained in comparison to the null model. *Indicate that the Holm-Bonferroni correction was applied. *β*/odds ratios and p-values for the variable in square brackets are calculated in comparison to the corresponding reference group. Bold text denotes statistical significance.

## 4. Discussion

The aim of this study was to determine the nutritional peaking strategies implemented by physique athletes and identify relationships between strategies and competitor characteristics. The results of this study indicate that competitors acutely manipulate their nutrition to enhance their onstage esthetic in accordance with previous research [14–16]. However, there was no association between competitor characteristics and peaking strategy length and the implementation of CHO-based strategies, while only water restriction and sodium load models were significant among the non-CHO-based strategies. Enhanced competitors were more likely to restrict water than natural, while sodium loading was more likely to be practiced by males and professionals in comparison to females and amateurs, respectively. Finally, sex predicted the range of daily CHO intakes during PW for both absolute and relative amounts.

### 4.1. Carbohydrate-based strategies

CHO manipulation strategies were prevalent within our sample where CHO back loading was the most popular strategy (45%). This finding is comparable to Chappell et al. [14] who noted that within the majority of their respondents (a more homogenous sample of bodybuilders) who manipulated CHO, restriction was followed by CHO loading. However, none of the present competitor characteristics predicted the implementation of CHO-based strategies which could be due to underpowered analyses from a small sample size. The lack of predictive power of the models, which range from only 2.3–5% of the variance explained (Tjur’s *R*^*2*^ = .023–.05) may highlight the variability of strategies among competitors or indicate unmeasured variables that may better predict the implementation of these strategies. In any case, appropriately exploring the relationships at play in this area is complex and warrants further research.

### 4.2. Water and electrolyte manipulation

Enhanced competitors are seemingly 2.5 and 3.1 times more likely to restrict water and sodium, respectively, compared to their natural counterparts. Despite the large odds ratios, these results should be interpreted with caution due to the wide confidence intervals as well as the non-significant and poor overall fit of the sodium restriction model (Tjur’s *R*^*2*^ = .088, binned residuals = 77%, *p* = .127). Nonetheless, these strategies may be implemented to minimize fluid retention, a common side effect of administering certain AAS (such as methandienone and nandrolone [24,25]) and other appearance enhancing drugs such as growth hormone [26,27]; however, more research is needed to determine what compounds and dosages cause water retention and to what degree they are used by physique athletes during contest preparation. Interestingly, both enhanced and natural competitors have been observed to restrict water and dietary sodium [14,28,29]. It has also been proposed that the temporal lag following manipulation of these variables could be leveraged to clear superfluous body water before homeostasis is reestablished, thereby enhancing the appearance of muscle [2]. Despite the theoretical potential to reduce fluid retention, the efficacy and safety of these strategies are under debate, and the effects of such practices on physique sport performance have not been empirically confirmed [1]. Ultimately, until future research is conducted, it is unknown whether there are net benefits or detriments in enhanced competitors when using such strategies. Further, it is likely that an individualized and carefully balanced approach needs to be found for such strategies to prove successful.

Interestingly, professional competitors were over fourfold more likely to load sodium than amateur competitors. This result might indicate an effort to maximize the effects of a “pump-up” routine [1,30]. While experimental evidence is required to determine the effects of increasing sodium intake before competition, it may plausibly result in acute increases in blood pressure and volume. Because these increases may potentially benefit performance in certain divisions, it is a speculative recommendation in previous reviews [1,30]. Although the magnitude of effect of sodium loading is unknown, more successful and higher-level competitors adopting this strategy may point to a degree of performance benefit. However, since very few professionals were sampled in this study (*N* = 16), and previous recommendations are predicated on empirically unconfirmed physiological reasoning, these results and recommendations should be interpretated and implemented with caution.

Males were also three times more likely to load sodium than females, which may be attributed to the judging criteria of divisions such as bikini. Bikini competitors, in contrast to male bodybuilders, are required to present less extreme muscle size and definition as well as vascularity [28]. Furthermore, while not significant, females had a higher likelihood of restricting water (OR = 2.04 [0.9, 4.69], *p* = .088), and despite poor model fit, females may be more likely to restrict sodium (OR = 3.67 [1.36, 10.39], *p* < .05). These results support the findings of Alwan et al. [29], who reported that 44% of their sampled female physique athletes manipulated sodium, and over 70% loaded and restricted water prior to competition. Accordingly, while the proportion of water loading was greater in females in our sample, the odds were non-significant (*p* = .269). Collectively, this may indicate that restricting water following loading is a common strategy used by female competitors to reduce subcutaneous water to enhance appearance with less concern for maintaining muscle size.

### 4.3. Peaking strategy length

While the number of days prior to competition when peaking began was not predicted by competitor characteristics, 66.7% of strategies commenced between 5 and 8 days prior to competition. This is in line with the recommendations of Escalante et al. [2] and is observed in case studies of peaking competitors, where manipulations began four to 6 days before competition [5,6,8,9]. These nutritional manipulation timeframes are similar to the traditional CHO loading protocols employed by endurance athletes [31].

Interestingly, a non-negligible number of competitors in our sample (8.8%) commenced their strategy 14 days away from competition, a value not previously observed in the literature. Post-hoc analysis of qualitative responses at this timepoint revealed that these competitors either increased energy intake slowly leading up to competition (i.e. CHO linear load), began restricting CHO severely, or sharply increased their water intake which was then restricted closer to competition. While a greater sample size and more detailed information is required to confirm why these competitors made such manipulations 2 weeks before competition, these responses indicate that different approaches were used. This may be due to these strategies being highly individualized and reactive, whereby competitors may have adjusted their strategy based on visual changes in appearance (i.e. muscle size and definition).

### 4.4. Range of daily carbohydrate intakes

When comparing the differences between the lowest and highest daily CHO intakes during PW for competitors, sex was a significant predictor, with females having a smaller disparity in both absolute and relative values. Thus, female competitors’ CHO intakes are more stable in PW compared to males who may engage in more extreme CHO manipulation strategies. This may reflect divisional differences, whereby a more extreme look is required for males and may compel them to implement depletion and loading phases. Similarly, competitors who achieved greater weight loss during contest preparation were more likely to have a greater difference between these values. This may be due to the requirements of certain divisions where competitors are required to achieve a very low level of adiposity, requiring very low energy (and thus CHO) intake, who then attempt to acutely increase muscle size through CHO loading strategies.

### 4.5. Strengths, limitations, and future directions

There are certain limitations that exist within this study. While we obtained a large sample size relative to previous surveys on PW practices [14,16], a greater sample size would have improved odds ratio and *β* accuracy, potentially reducing type II errors, and enhancing the generalizability of the results. Additionally, a larger sample size potentially captures more competitors of different divisions, whereby the interactions between competitor division and peaking strategy could be explored further. The generalizability of the results is also likely constrained by the way the survey was distributed. As the survey was mostly advertised by prominent social media exercise science communicators, it is likely that the approaches of the participants may have been influenced by who they follow on social media. While effort was made for the survey to be advertised by a range of social media users, this may have led to the survey only capturing a certain segment of competitors. Finally, as the number of eligible competitors who were not reached or did not respond to the survey is unknown, generalizing the results of this survey is difficult.

This was a large survey with many questions where 160 participants completed the nutrition section of the survey; however, only 104 completed the survey in full. This may hold implications on the validity of the responses in addition to common issues with surveys (biased or incomplete recollections, mostly true responses with some fabricated details, or entirely false responses) [19]. To counteract these limitations, the participants were informed that the survey was anonymous before commencing, which may have reduced deliberate false responses. Further, responses were screened for unambiguously false entries to minimize inaccuracies and false information (e.g. improbable/random combination of peaking strategies). Based on our low completion rate, we recommend future researchers build on our findings with more targeted approaches. Using a simpler but concentrated survey to increase the number of responses on more specific topics might be reasonable trade-off for a less encompassing view of PW strategies.

It is important to acknowledge the inherent variability of peaking strategies, as the sheer number of interrelated variables makes it difficult to capture all aspects of PW with robust models. Nevertheless, exploring other variables that may predict PW strategies is a valuable avenue of investigation. Finally, while this survey captured both nutrition and training variables during PW, their interactions were not evaluated for the purpose of this study and require further exploration.

## 5. Conclusions

The relationships between competitor characteristics and nutritional PW strategies were examined with a series of regression models. No significant predictors were found for CHO-based PW strategies, nor for PW strategy length. In comparison to females, males were more likely to load sodium and have greater differences between their highest and lowest PW CHO intakes, and while not significant, females had a higher likelihood of restricting water. Finally, enhanced competitors were more likely to restrict water than their natural counterparts and professional competitors more likely to load sodium than amateurs. This study provides further insight on the relationship between physique athlete characteristics and their nutritional peaking strategies which display high degrees of complexity and individualization. Nevertheless, our results should be interpreted and generalized with caution pending future work with larger samples allowing more confident assertions.

## Supplementary Material

Supplemental Material

## Data Availability

The dataset analyzed for this research can be accessed on the Open Science Framework repository (https://osf.io/9ghfz/).

## Geolocation information

This research involved an online survey where the geolocation of participants was not recorded.

## Health and safety

As this research only involved the completion of an online survey, there were no risks to the health and safety of participants. Further, participants were aged 18 years or older, and as no identifiable information was collected, the anonymity of all participants was protected.
