# Supplementary material for: An examination of the associations between nutritional peaking strategies in physique sport and competitor characteristics
Source: J Int Soc Sports Nutr. 2024 Jul 15;21(1):2377178. doi: 10.1080/15502783.2024.2377178 (PMC11251432; doi:10.1080/15502783.2024.2377178)
Supplement: Supplemental Material [file RSSN_A_2377178_SM1555.docx]

**Definitions of Nutritional Peaking Strategies Presented to Participants**

No peaking strategy: No major change from previous week(s), similar relative total intakes of total energy, protein, carbohydrates, fat, water, sodium, potassium, and alcohol.

Carbohydrate back-load protocol: Begins with a carbohydrate depletion phase (typically two to four days) before loading over the next one to three days prior to competition. An example of this protocol is depicted below. If you maintain caloric intake during depletion by increasing fat consumption, please select the “fat load” option as well.


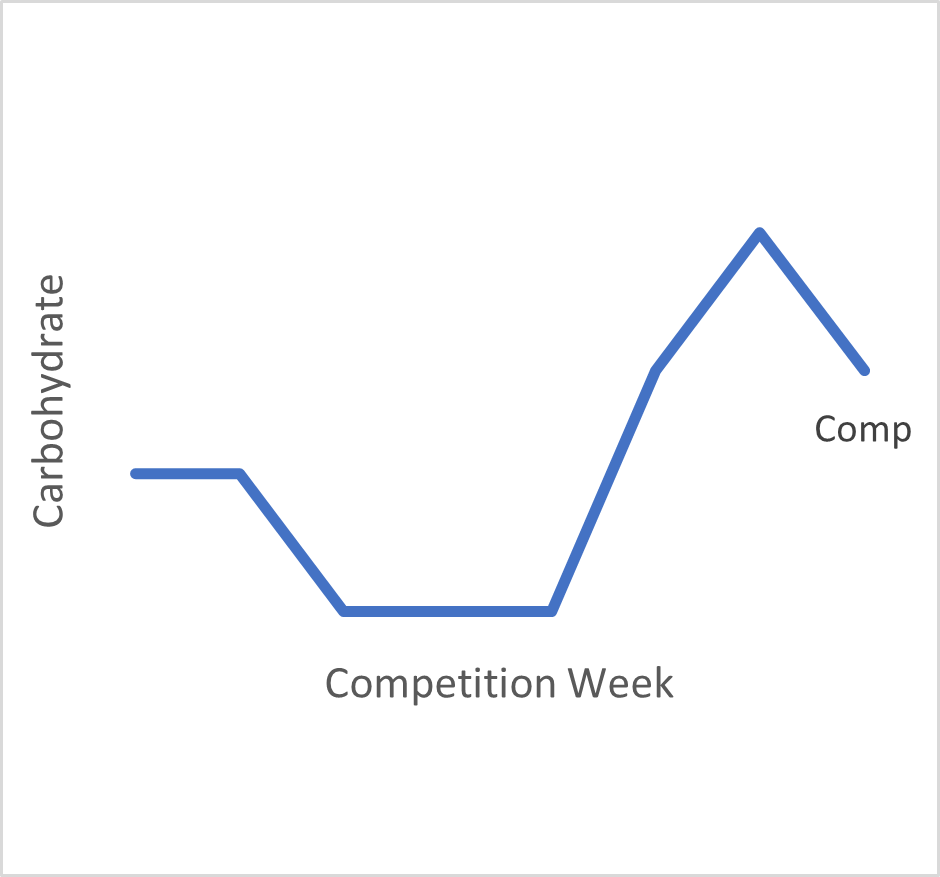


Carbohydrate mid-load protocol: Carbohydrate is low at the start of the week and is gradually increased prior to loading mid-week (typically Wednesday and Thursday). Intake is tapered for the final days prior to competition. An example of this protocol is depicted below.


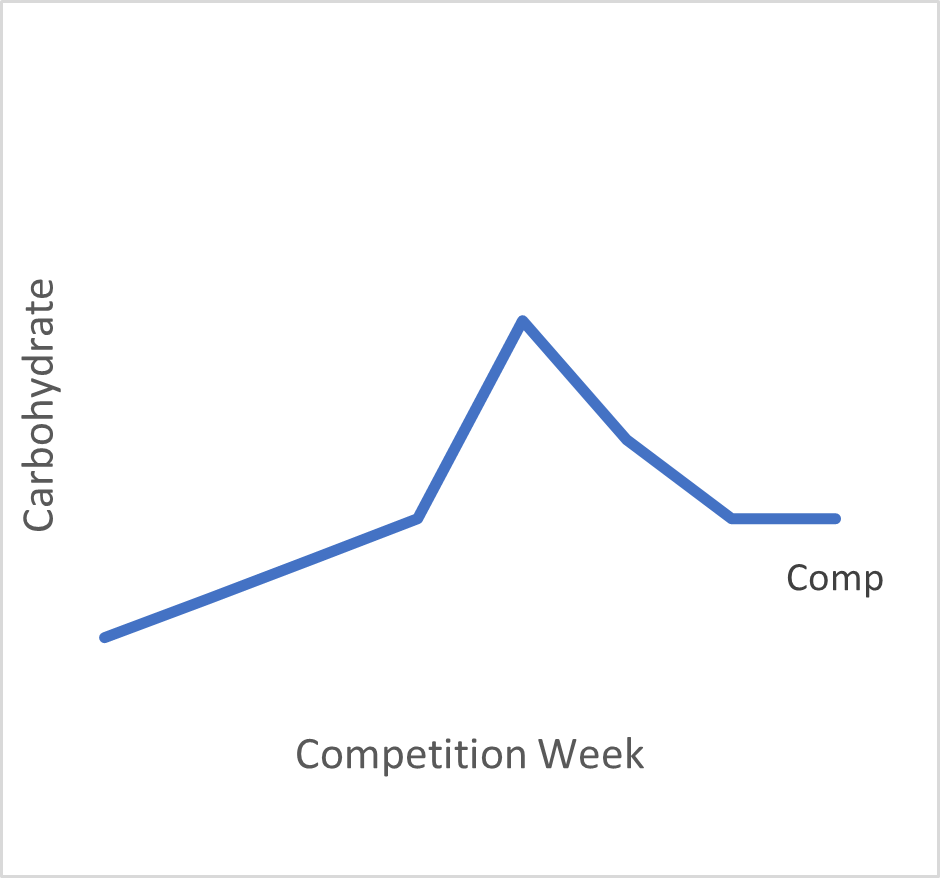


Carbohydrate front-load protocol: Carbohydrate is loaded at two periods, once at the start of the week and is gradually decreased until another load later in the week. An example of this protocol is depicted below. If you maintain calorie intake during depletion by increasing fat, please select the “fat-load” option as well.


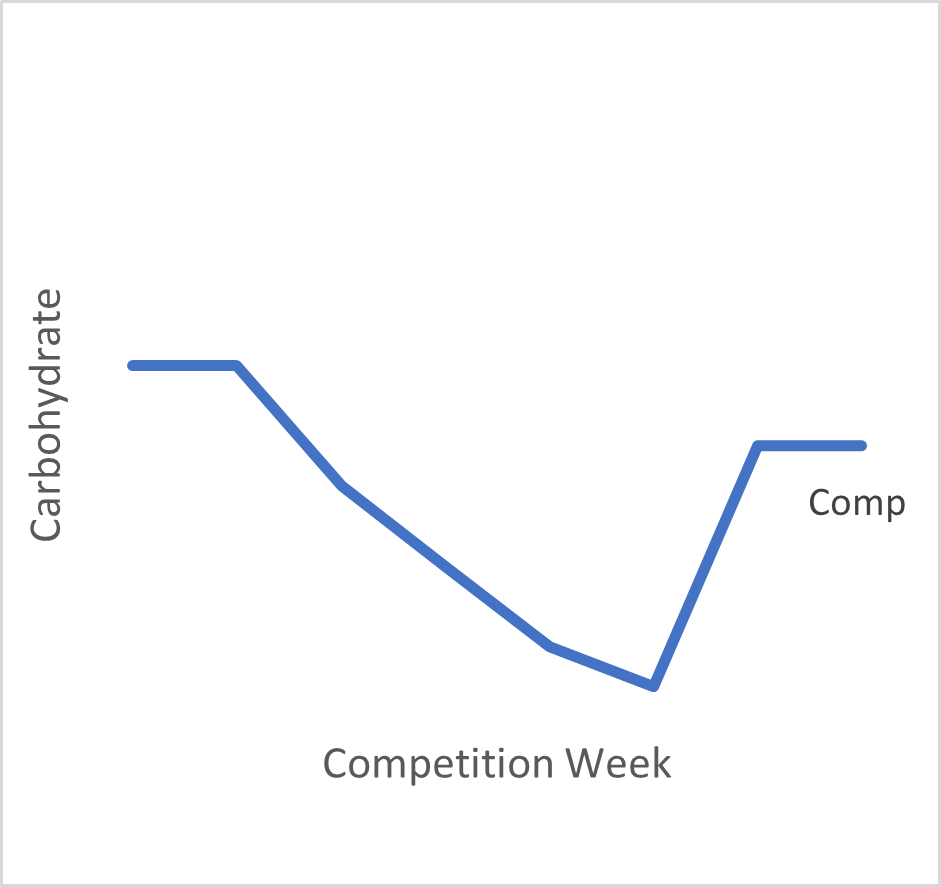


“Linear” carbohydrate load: Commonly known as “Eating up” into the show. Involves multiple weeks of an increase in energy and/or carbohydrates. An example of this protocol is depicted below.


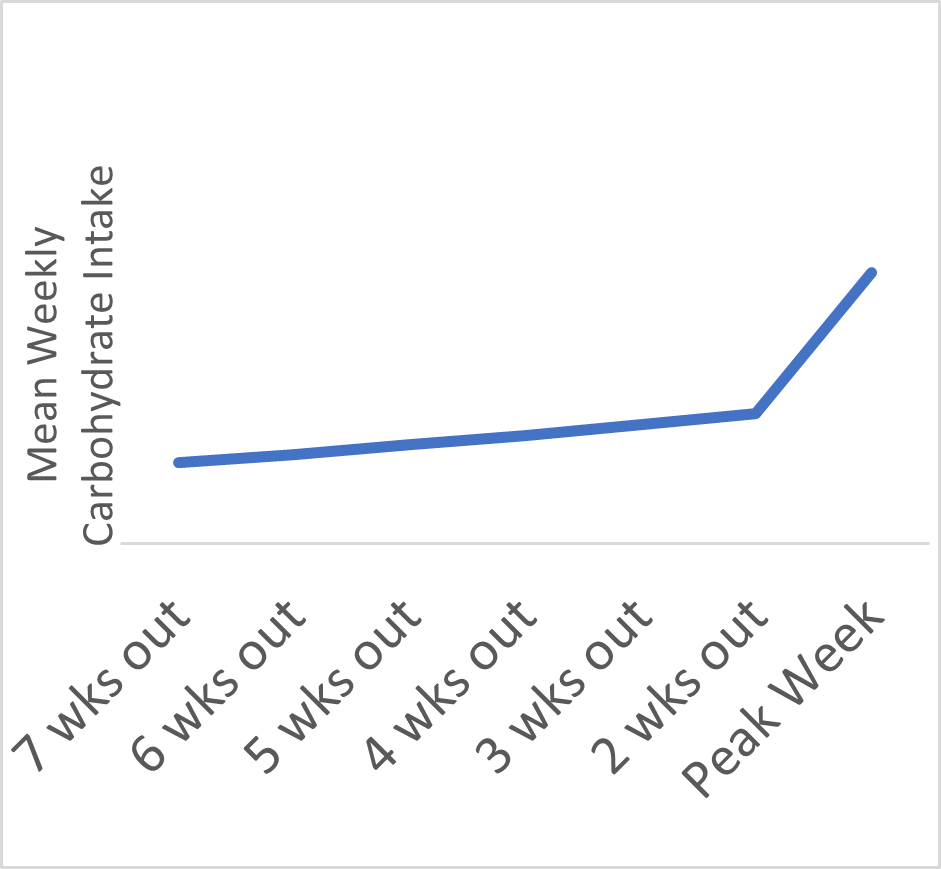


Carbohydrate restriction: Further restriction of carbohydrates within the final week of competition relative to prior weeks without a refeed or subsequent loading. An example of this protocol is depicted below.


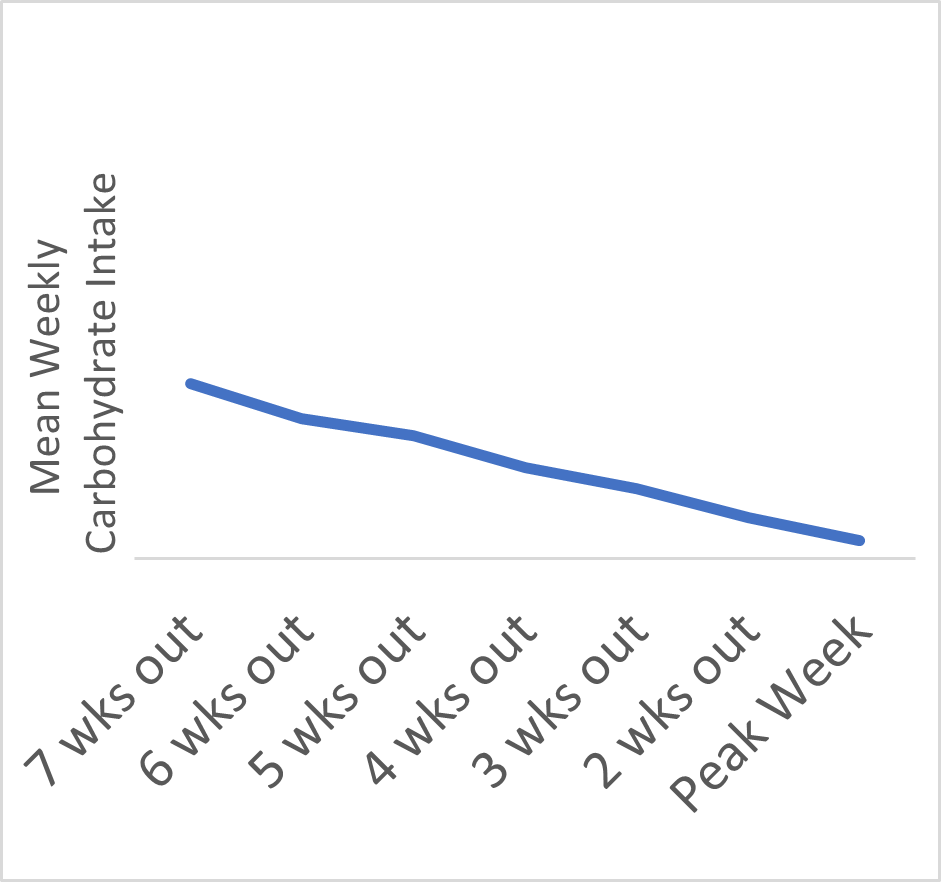


Fat load: An increase in fat intake for at least one day during peak week.

Water load: Additional water consumption on at least one day relative to prior weeks. If you load **AND** restrict water, please tick the “water load” option as well.

Water restriction: Reduced water consumption on at least one day relative to prior weeks. If you load **AND** restrict water, please tick the “Water load” option as well.

Sodium load: Additional sodium consumption on at least one day relative to prior weeks.

Sodium restriction: Restriction of sodium consumption on at least one day relative to prior weeks. If you restrict sodium **AND** load potassium, please tick the “Potassium load” option as well.

Potassium load: Increased potassium consumption on at least one day relative to prior weeks. If you restrict sodium **AND** load potassium, please tick the “Sodium restriction” option as well.
